# Influence of Individual Differences on the Calculation Method for FBG-Type Blood Pressure Sensors

**DOI:** 10.3390/s17010048

**Published:** 2016-12-28

**Authors:** Shouhei Koyama, Hiroaki Ishizawa, Keisaku Fujimoto, Shun Chino, Yuka Kobayashi

**Affiliations:** 1 Institute for Fiber Engineering, Shinshu University, 3-15-1 Tokida, Ueda City, Nagano 386-8567, Japan; zawa@shinshu-u.ac.jp; 2 Department of Clinical Laboratory Sciences, School of Health Sciences, Shinshu University, 3-1-1 Asahi, Matsumoto City, Nagano 390-8621, Japan; keisaku@shinshu-u.ac.jp; 3 Graduate School of Science and Technology, Shinshu University, 3-15-1 Tokida, Ueda City, Nagano 386-8567, Japan; 15fm218d@shinshu-u.ac.jp (S.C.); 16fs112j@shinshu-u.ac.jp (Y.K.)

**Keywords:** FBG, blood pressure, acceleration pulse wave, individual difference, Partial Least Squares Regression

## Abstract

In this paper, we propose a blood pressure calculation and associated measurement method that by using a fiber Bragg grating (FBG) sensor. There are several points at which the pulse can be measured on the surface of the human body, and when a FBG sensor located at any of these points, the pulse wave signal can be measured. The measured waveform is similar to the acceleration pulse wave. The pulse wave signal changes depending on several factors, including whether or not the individual is healthy and/or elderly. The measured pulse wave signal can be used to calculate the blood pressure using a calibration curve, which is constructed by a partial least squares (PLS) regression analysis using a reference blood pressure and the pulse wave signal. In this paper, we focus on the influence of individual differences from calculated blood pressure based on each calibration curve. In our study, the calculated blood pressure from both the individual and overall calibration curves were compared, and our results show that the calculated blood pressure based on the overall calibration curve had a lower measurement accuracy than that based on an individual calibration curve. We also found that the influence of the individual differences on the calculated blood pressure when using the FBG sensor method were very low. Therefore, the FBG sensor method that we developed for measuring the blood pressure was found to be suitable for use by many people.

## 1. Introduction

Japan is an aging society [1], and the Japanese people understand the importance of monitoring their health on a daily basis. Therefore, there is a high need for wearable health monitoring sensors. Our research is focused on vital sign measurement sensors using fiber Bragg grating (FBG) sensors, which are high precision optical fiber type strain sensors that use a diffraction grating that is sensitive to the optically induced refractive index change in optical fibers. The diffraction grating is the sensing component in the FBG sensor, and detects the strain by applying the diffraction grating to measure the Bragg wavelength shift. The FBG sensor is used in various fields as strain sensor. Tam et al. presented a FBG sensors for structural and railway applications [2,3,4]. In addition, the research of vital sign measurement using the FBG sensor are reported [5,6,7].

In a previous study, we measured various vital signs using an FBG sensor [8,9,10]. We found that the peak interval that could be measured using the FBG sensor signal was very similar to the R-R interval (RRI) in an electrocardiogram measurement. Therefore, the signals received by the FBG sensor did indicate the heart rate. By performing various calculations on the signals from the FBG sensor, the pulse rate and respiration rate could be measured. The blood pressure, which is the focus of this paper, is another example of a vital sign measurement. We first measured the blood pressure using two FBG sensors that were located at different distances from the heart of the subject [11,12]. There was a time difference in the peaks from the two FBG sensor signals because they were different distances from the heart. The blood pressure was calculated based on this time difference. However, this method had a disadvantage in that the subjects were not able to move, due to the two sensors on the surface of their body. This measurement method does not use wearable sensors. As an alternative, we calculated the blood pressure based on the signal measured at a single FBG sensor, and then constructed a calibration curve from the measured pulse wave signal data using the partial least squares (PLS) regression method.

The purpose of this paper is to examine the influence of individual differences on the calculation method. In the previous study, the blood pressures were calculated using individual calibration curves for each subject in order to eliminate the possibility of individual differences influencing the results. However, as it takes a great deal of effort to construct a calibration curve for each subject, it became desirable to construct a calibration curve that is applicable to all subjects. To accomplish this objective, it is important to first examine the influences of individual differences in the blood pressure calculation method. In this paper, we describe the results of our study into the influence of individual differences on calculated blood pressure results using two analytical methods to generate an overall calibration curve and an individual calibration curve.

## 2. Measurement Principle

### 2.1. Measurement System of the FBG Sensor

**Figure 1:**
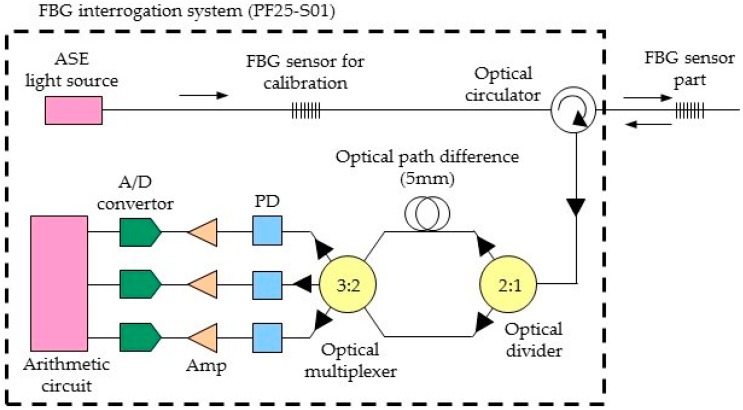
The block diagram of the interrogator and FBG sensor.

Our experimental setup includes a PF25-S01 FBG interrogator system (Nagano Keiki Co., Ltd., Ota-ward, Tokyo, Japan), and an ASE light source that emitted a near-infrared light with a wavelength of 1525–1570 nm. Figure 1 shows a block diagram of the interrogator and FBG sensor. 

Broadband near infrared light is emitted from the ASE light source, and the light reflected by the FBG sensor. The wavelength of the detected light is determined by the Bragg equation for reflected light:

(1) $$\lambda_{Bragg}=2n_{eff}\Lambda$$

where λ_Bragg_ is the Bragg wavelength, n_eff_ is the refractive index of the optical fiber core, and Λ is the diffraction grating spacing. The refractive index of the optical fiber (n_eff_) is fixed. When pressure is applied to the sensor, the diffraction grating spacing changes, which causes the detected Bragg wavelength to also change in according with Equation (1). The Bragg wavelength is detected by an InGaAs detector through the medium of the Mach-Zehnder interferometer. In the Mach-Zehnder interferometer, the one optical fiber length is about 3 mm longer, the refractive index is 1.5, therefore the optical path difference is about 5 mm. The homodyne detection method using the Mach-Zehnder interferometer detects the shift length of Bragg wavelength as interference phase shift [13,14], as follows:

(2) $$\Delta \phi =\frac{2\pi n_{eff}d}{{\lambda_{Bragg}}^{2}}\Delta \lambda_{Bragg}$$

where Δϕ is the phase shift, and d is the optical path difference.

The advantage of this method is the wavelength measurement resolution is smaller than other methods. First, in order to calibrate the influence of temperature etc., FBG sensor is measured without being installed on the pulsate point (calibration measurement). After the calibration measurement, the pulse wave signal is measured by installing the FBG sensor on the pulsate point. After the Bragg wavelength is passing through the Mach-Zehnder interferometer, which outputs by the three detectors. The output V_n_ is given by:

(3) $$V_{n}=\alpha_{n}(C+\cos(\phi (t)+\frac{2\pi (n_{eff}-1)}{3}))$$

where α and C represents the amplitude of the three signals and offset of the signals. It is converted into an electric signal by the detectors, which through the amplifier, and converted to a digital signal by the AD converter. The phase angle is demodulated by the arithmetic circuit, and wavelength shift is computed. The phase angle calculation equation of three signals with different phases is expressed by Equation (4) using trigonometric functions:

(4) $$\tan\phi (t)=\frac{\sqrt{3}(\alpha_{3}V_{2}-\alpha_{2}V_{3})}{\left(\alpha_{3}V_{2}+\alpha_{2}V_{3}-2\alpha_{2}\alpha_{3}V_{1}\right)}$$

The amplitudes and offset are adjusted to 1 by the calibration measurement, and Equation (4) is transformed to Equation (5):

(5) $$\tan\phi (t)=\frac{\sqrt{3}(V_{2}-V_{3})}{\left(V_{2}+V_{3}-2V_{1}\right)}$$

Equation (5) is transformed to Equation (6) by the arctangent function:

(6) $$\phi (t)=\arctan\frac{\sqrt{3}(V_{2}-V_{3})}{\left(V_{2}+V_{3}-2V_{1}\right)}$$

The wavelength shifts of Δλ is calculated by substituting ϕ in Equation (6) into Equation (2). Therefore, the pressure of the FBG sensor part is detected by measuring the Bragg wavelength shifts.

### 2.2. Measurement of the Pulse Wave Signal by the FBG Sensor

An FBG sensor is a type of strain sensor. When an FBG sensor is installed in a location where there is no distortion, then no signal is measured. If an FBG sensor is positioned to measure blood flow, and the blood flow at that point is flowing at a constant rate, then the Bragg wavelength does not change. On the other hands, when the speed of the blood flow changes, the Bragg wavelength also change. The rate at which the speed of the blood flow changes shows the acceleration of the blood flow. That is, the signal measured at the FBG sensor is the acceleration pulse wave signal. 

**Figure 2:**
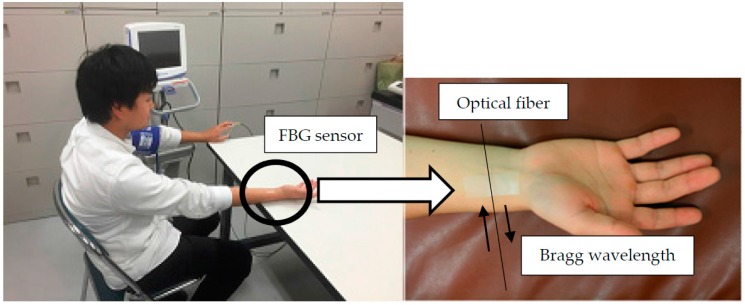
Experimental image of the pulse wave signal being measured by the FBG sensor.

There are several pulse points on the surface of the human body, including the wrist, neck, temple, and ankle. When an FBG sensor is affixed using medical tape to one of these points, as shown in Figure 2, the pulse wave signal can be measured. In the figure, the subject is sitting, and the FBG sensor is positioned on their right wrist. The FBG sensor are installed in the same orientation. The infrared light passes towards the orientation of the subject’s body. Therefore, there is no influence for the waveform by the orientation of FBG sensor. In our study, the participants included three elderly persons plus several healthy subjects in their 20s. All measurement conditions were the same for each subject, the sampling rate of the FBG interrogator system was 10 kHz, and the measurement time was 20 s. The measured pulse wave signal is processed by the band-pass filter (0.5–5 Hz). This band-pass filter processing signals used as a measured pulse wave signals. The waveform of the measured pulse wave signal was then validated.

### 2.3. Blood Pressure Measurement Calculation Method

The pulse wave signal is caused by pressure that propagates to the surface of the body from the inside of the blood vessel due to the blood flow. The pulse wave signal includes information that can be used to determine the blood pressure. Consequently, we expect to be able to calculate the blood pressure by analyzing the pulse wave signal [15,16]. In the field of infrared spectroscopy measurements, there are well developed methods for calculating sample information using measured spectra and chemometrics [17,18,19,20,21]. The spectrum is the collection of light intensities of each wavelength, and the pulse wave signal is a spectrum-like signal that consists of the collection of the Bragg wavelength shifts at each sampling point. The information about the blood pressure in the pulse wave signal will be calculated using chemometrics.

PLS is a method used in chemometrics [22,23,24,25] that involves multivariate analysis, and is similar to the principal component regression (PCR) method. The principal component factor is calculated using an explanatory variable and an objective variable that is assumed to include an error. This point is different from the PCR. In the PCR method, the objective variable is assumed to have no error. In this paper, since the measurement value at the electronic blood pressure monitor including error is the objective variable, PLS is used for the calibration curve construction method. The principal component factor (PLS factor) is then calculated and the regression equation is built. The new objective variables are calculated by using this regression equation. A new PLS factor is calculated with the new objective variables and explanatory variables, and the regression equation and the objective variables are recalculated by adding this PLS factor. By repeating this calculation, as the number of PLS factors increases, a regression equation that makes the error smaller is built. However, if the number of PLS factors is increased too much, the standard error of prediction (SEP) is larger. (over fitting). The optimum PLS factors number is verified by the leave-one-out method when computing a new PLS factor. The prediction residual error sum of squares (PRESS) in the model before and after adding the PLS factor is verified by the F-test. When the difference between each PRESS is significant, PLS factor is added. When it is not significant, the regression equation of the model with a smaller number of PLS factors is selected as the calibration curve.

The blood pressure is calculated from the FBG sensor signal using two processes. The first process builds a calibration curve to calculate the blood pressure. To reduce the measurement error, it is important to build a high accuracy calibration curve. In the second process, the measured FBG sensor signals are substituted into the calibration curve before the blood pressure is calculated. Once a high accuracy calibration curve has been built, only the second process is required to measure the blood pressure. 

**Figure 3:**
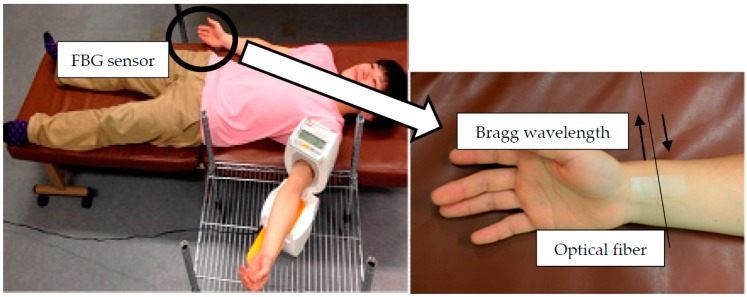
Experimental image of blood pressure measurement by the FBG sensor.

When measuring the blood pressure of the subject, as shown in Figure 3, the FBG sensor is positioned on their right wrist. The reference blood pressure is simultaneous measured by an electronic sphygmomanometer (PVM-2701, Nihon Kohden Co., Ltd., Shinjuku-ward, Tokyo, Japan) in the left upper arm. The measurement is performed while the subject is lying face up. The pulse wave signal of the subject is measured at 1 min intervals for 20 s. The subjects consisted of three males in their 20s.

The pulse wave signal and reference blood pressure were measured 50 times to create the calibration data set. The signals were signal processed, each peak was extracted, and then the peaks were averaged and normalized. In the normalized signal, the height of the clipping position was “1” and the minimum point was “0”. The normalized signal and reference blood pressure were used as the explanatory and objective variables, respectively, and then the calibration curve was constructed using PLS regression. At a later date, the same subject was again measured 25 times in order to validate the data.

**Figure 4:**
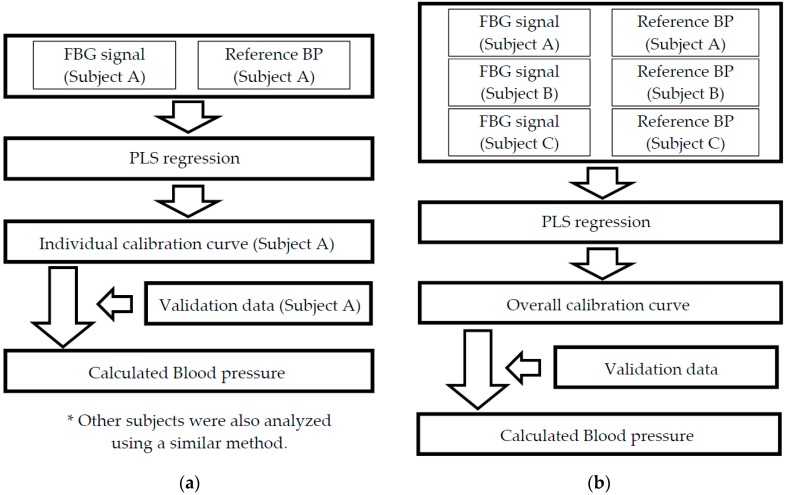
The block diagram of each calibration curve constructing method: (**a**) the method of constructing an individual calibration curve; and (**b**) the method of constructing the overall calibration curve.

The best calibration curve for calculating blood pressure is the calibration curve applicable to anyone. As this calibration curve requires data sets measured by various subjects, the influence of individual differences is included. If this influence is large, the measurement error of the calculated blood pressure is larger by using the overall calibration curve. On the other hand, if calibration curves for each subject are built, the influence of individual differences will be eliminated, however a lot of effort is required when many people use the individual calibration curve. The individual calibration curve method is not suitable for many people to use by the blood pressure calculation method proposed in this paper. Therefore, in order to verify the influence of individual difference when building the overall-calibration curve, the measurement accuracy of the calculated blood pressure by using the overall-calibration curve and the individual-calibration curve must be compared. There were two methods used to build the calibration curve, and Figure 4 shows a block diagram of each. The calibration and validation data sets used to build the calibration curves were the same. First, a calibration curve was built using 50 FBG signals and the reference blood pressure data from each subject (individual calibration curves A, B, and C). The blood pressure of subject A was calculated by substituting the verification data A for individual calibration curve A. Individual calibration curve A did not include data for subjects B and C. Therefore, individual differences were not able to influence the validation blood pressure results measured using individual calibration curve A. The same analysis method was also used for subjects B and C. Second, a calibration curve was built using 150 data points (50 measurements × 3 individuals). This was the overall calibration data set of all subjects (i.e., the overall calibration curve). The blood pressure of subject A was calculated by substituting the verification data A for the overall calibration curve. The overall calibration curve includes data from subjects B and C. Therefore, individual differences are able to influence the validation blood pressure results through the overall calibration curve. The influence of the individual differences was verified based on the calculated results from the two methods.

## 3. Measurement Results

### 3.1. Results of Pulse Wave Signal Measurement by the FBG Sensor

**Figure 5:**
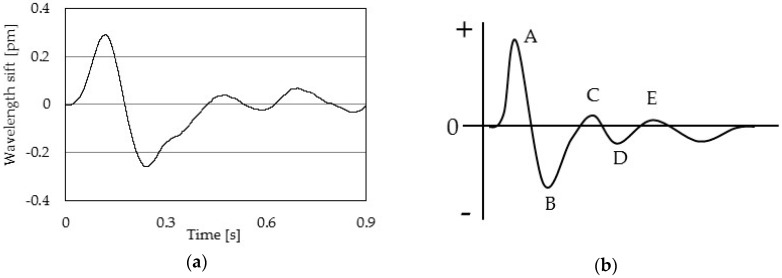
The pulse wave signals: (**a**) pulse wave signal measured by the FBG sensor; (**b**) pulse acceleration pulse wave of the second derivative of the pulse wave signal.

Figure 5a shows a one pulse wave signal measured by an FBG sensor in a healthy subject, and Figure 5b shows a one pulse acceleration pulse wave of the second derivative of the pulse wave signal [26]. 

**Figure 6:**
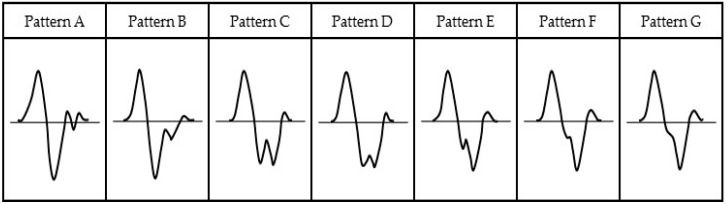
Seven patterns of the acceleration pulse wave.

The pulse wave signal has a five peaks (denoted A to E in the figure). Peak A is an early systolic positive wave, and peak B is an early systolic negative wave [27]. These peaks show a driving pressure wave caused by the ejection of blood due to a heart contraction. Peak C is a late systolic reincreasing wave, and peak D is a late systolic redecreasing wave [27]. These two peaks show the driving pressure wave reflected from the peripheral blood vessels. Peak E is an early diastolic positive wave [27]. This pulse wave signal is very similar to the acceleration pulse wave of the second derivative wave of the fingertip volume pulse wave signal. The acceleration pulse wave is divided into seven patterns that depend on age and whether or not the subject has vascular disease. Figure 6 shows the seven patterns in the acceleration pulse wave [28]. Pattern A is the acceleration pulse wave of a healthy person. As the age increases, the acceleration pulse wave will change from pattern B to E. If patterns F and G are observed, the subject is suspected of having arteriosclerosis. If the pulse wave signal indicates an acceleration pulse wave, waveform of pulse wave signal of the elderly will change.

**Figure 7:**
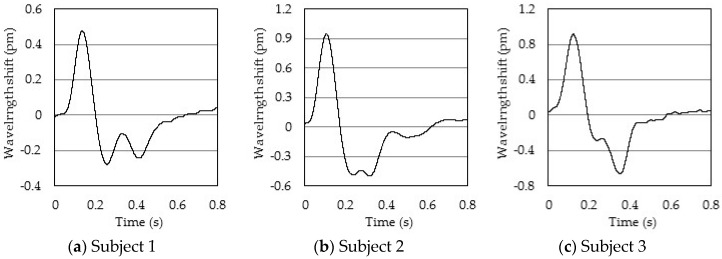
The measured pulse wave signals of three elderly persons: (**a**) Subject 1; (**b**) Subject 2; (**c**) Subject 3.

Figure 7 shows the measured pulse wave signals of three elderly individuals. The pulse wave signal of subject 1 is similar to pattern C. Similarly, subject 2 is similar to pattern D, and subject 3 is similar to pattern F. The pulse wave signals of an elderly person are different than those of a healthy person. Based on these results, the pulse wave signal measured by the FBG is an acceleration pulse wave in the subject. Since the sampling rate is high, we are able to detect small changes in each pattern. As a result, the pulse wave signal measured by the FBG sensor can be used for pulse wave diagnosis in clinical practice.

### 3.2. The Influence of Individual Differences in the Blood Pressure Calculated Using the Calibration Curve Method

**Figure 8:**
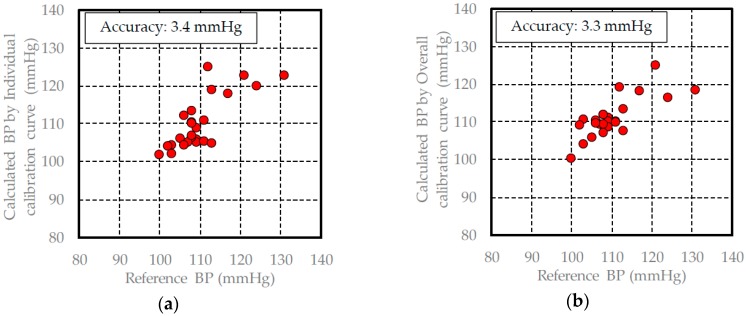
Validation results of the blood pressure in subject A: (**a**) calculated blood pressure results using the individual calibration curve; (**b**) calculated blood pressure results using the overall calibration curve.

**Figure 9:**
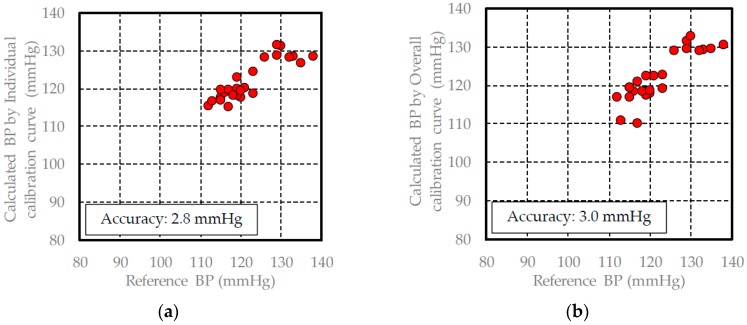
Validation results of the blood pressure in subject B: (**a**) calculated blood pressure results using the individual calibration curve; (**b**) calculated blood pressure results using the overall calibration curve.

**Figure 10:**
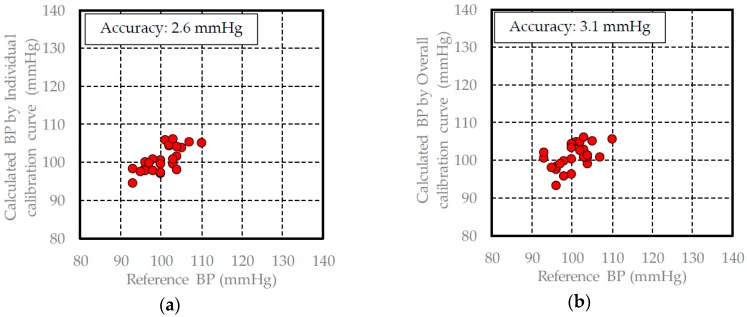
Validation results of the blood pressure in subject C: (**a**) calculated blood pressure results using the individual calibration curve; (**b**) calculated blood pressure results using the overall calibration curve.

In Section 3.1, the pulse wave signal measured by the FBG sensor is similar to the acceleration pulse wave signal, and each peak includes information regarding the driving pressure from the heart. Therefore, the blood pressure can be calculated from the driving pressure information. The calibration and validation data for each healthy subject are shown in Table 1 and Table 2, and the individual and overall calibration curves are built using the PLS method and the calibration data of each subject. The results of building each calibration curve are shown in Table 3. The individual calibration curves of all subjects were built to have a high measurement accuracy and high correlation coefficient. The blood pressure was calculated by substituting the validation data for each of the subjects into these calibration curves. The results of the calculated blood pressure when the individual calibration curves were used are shown in Figure 8, Figure 9 and Figure 10.

The measurement accuracy when calculating the blood pressure using an individual calibration curve for a subject was approximately 3 mmHg, while the measurement accuracy of a commercial blood pressure meter is about 5 mmHg. Therefore, the blood pressure measuring method that we developed using the FBG sensor provides a high degree of accuracy. With regard to the overall calibration curve, the correlation coefficient was 0.93, and the measurement accuracy was 4.3 mmHg. At 0.93, the correlation coefficient for the results using the overall calibration curve was better than that for the results using the individual calibration curves. However, the measurement accuracy of the overall calibration curve was lower than the results using the individual calibration curves. This finding was verified using the average blood pressure in the calibration data. The average blood pressure range based on the calibration data for each subject was approximately ±10 mmHg, while the actual average blood pressure of each subject was approximately 100, 110, and 120 mmHg. When the data from the three subjects is combined, the measured blood pressure range was 90–130 mmHg, while the average blood pressure was 111.8 mmHg ± 20 mmHg. Therefore, the data from the three subjects was consistent, and the combined calibration curve resembled a straight line where Y = X. Based on the above reasoning, in this case, the correlation coefficient was higher and the measurement accuracy was lower. 

The measurement accuracy of the calculated blood pressure using the overall calibration curve was 3.1 mmHg. This measurement accuracy was lower than the measurement accuracy when the individual calibration method was used for subjects B and C. This may be due to the influence of individual differences because the measurement accuracy of the calculated blood pressure changed depending on whether the overall or individual calibration curves were used. However, the measurement accuracy when the overall calibration curve was used was less than 5 mmHg. The influence of individual differences in the overall calibration curve in the calculated method was small and had little effect on the measurement accuracy. The results of the diastolic blood pressure measurements also demonstrated a similar tendency. Based on these results, in the developed blood pressure measurement method, the influence of individual differences was found to be small. Therefore, we were successfully able to build an overall calibration curve that is widely applicable.

## 4. Conclusions

In order to verify our method of measuring blood pressure using an FBG sensor, we compared our results to the measured pulse wave signal and the calculation method for determining the blood pressure:

- The pulse wave signal measured by the FBG sensor is similar to the acceleration pulse wave signal.
- The waveform of the measured pulse wave signal differs between healthy and elderly individuals.
- The overall calibration curve has the influence of individual differences, because the measurement accuracy of the calculated blood pressure was lower than the measurement accuracy using the individual calibration method.
- The measurement accuracy of the calculated blood pressure using the overall calibration curve is approximately 3 mmHg.

By changing the analysis method in the same measurement signal, an FBG sensor can measure the pulse rate, respiratory rate, and human stress level in addition to the blood pressure. 

In the future, some challenges will be encountered when developing a multi-vital sign sensor based on an FBG sensor. In this study, the blood pressure was calculated from the pulse wave signals measured by the FBG sensor; however, this method has not been proved theoretically. To do this, we must prove the nature of the relationship between the acceleration pulse wave signal and the blood pressure. Then, it will be necessary to measure the blood pressure in subjects of various ages because the waveform of the pulse wave signal changes with the age of the subject. In addition, the typical method of processing the pulse wave signal also needs to be changed. Furthermore, the typical calibration curve constructed using the PLS method must be changed. In this experiment, we developed a curve that can be applied to healthy subjects. However, for the elderly and patients with hardening of the arteries, a suitable calibration curve that accounts for the waveform of the pulse wave signal is required.

In the physical device, the size of the FBG interrogator system should be less than 50 mm. Currently, the PF25-S01 interrogator system (the light source and the Mach-Zehnder interferometer type optical receiver system) has a width, depth, and height of 330, 230 and 105 mm, respectively. In order to reduce the size of the FBG interrogator system, a detection method other than the Mach-Zehnder interferometer type is required. In our research, we designed a smaller FBG interrogator system. Next, smart textiles need to be fabricated that include optical fibers and FBG interrogator system. When wristband type FBG sensing system have been developed, then they can be used to measure the vital signs. This type of wearable FBG sensing system can be used for long-term continuous measurements. If these challenges can be overcome, then it will be possible to develop wearable sensors that can measure multiple vital signs.

## Figures and Tables

**Table 1:** Calibration data set of each subject.

Subject	Samples	Max (mmHg)	Min (mmHg)	Avg (mmHg)
A	50	125	100	111.3
B	50	136	113	123.1
C	50	111	93	100.9
Overall	150	136	93	111.8

**Table 2:** Validation data set of each subject.

Subject	Samples	Max (mmHg)	Min (mmHg)	Avg (mmHg)
A	25	131	100	110.1
B	25	138	112	122.2
C	25	110	93	100.4

**Table 3:** Results of each individual calibration curve and overall calibration curve.

Calibration curve	R	Accuracy (mmHg)
Individual Subject A	0.82	4.2
Individual Subject B	0.89	3.0
Individual Subject C	0.67	3.1
Overall	0.93	4.1

## References

[1] Cabinet Office, Government of Japan Situation on Aging, Annual Report on the Aging Society 2015. http://www8.cao.go.jp/kourei/english/annualreport/2015/pdf/c1-1.pdf.

[2] Tam H.Y., Liu S.Y., Guan B.O., Chung W.H., Chan T.H., Cheng L.K. (2005). Fiber bragg grating sensors for structural and railway applications. Proc. SPIE.

[3] Wei C.L., Lai C.C., Liu S.Y., Chung W.H., Ho T.K., Tam H.Y., Ho S.L., McCusker A., Kam J., Lee K.Y. (2014). A fiber bragg grating sensor system for train axle counting. IEEE Sens. J..

[4] Wei C.L., Xin Q., Chung W.H., Liu S.Y., Tam H.Y., Ho S.L. (2012). Real-time train wheel condition monitoring by fiber bragg grating sensors. Int. J. Distrib. Sens. Netw..

[5] Hao J., Jayachandran M., Kng P.L., Foo S.F., Aung P.W., Cai Z. (2010). FBG-based smart bed system for healthcare applications. Front. Optoelectron..

[6] Spillman W.B., Mayer M., Bennett J., Gong J., Meissner K.E., Davis B., Claus R.O., Muelenaer A.A., Xu X. (2004). A ‘smart’ bed for non-intrusive monitoring of patient physiological factors. Meas. Sci. Technol..

[7] Elsarnagawy T., Haueisen J., Farrag M., Ansari S.G., Fouad H. (2014). Embedded fiber bragg grating based strain sensor as smart costume for vital signal sensing. Sens. Lett..

[8] Kawamura M., Ishizawa H., Sato S., Koyama S. Application to vital signs by fiber bragg grating sensing. Proceedings of the SICE Annual Conference 2011 Final Program and Papers.

[9] Miyauchi Y., Ishizawa H., Koyama S. The pulse rate measuring system which use FBG sensors. Proceedings of the 13th International Symposium on the Science and Technology of Lighting.

[10] Sato S., Ishizawa H., Hattori A., Miyauchi Y. Study of fixed points in pulse rate measurement by FBG sensor. Proceedings of the SICE Annual Conference 2012 Final Program and Papers.

[11] Miyauchi Y., Ishizawa H., Koyama S., Sato S. Verification of the systolic blood-pressure measurement principle by FBG Sensors. Proceedings of the SICE Annual Conference 2012 Final Program and Papers.

[12] Takagi T., Ishizawa H., Niimura M., Koyama S., Miyauchi Y., Katsuragawa Y. Basis study on systolic blood pressure measurement by using FBG sensors. Proceedings of the International Symposium on Fiber Science and Technology 2014.

[13] Yoshino T., Sano Y., Ota D., Fujita K., Ikui T. (2016). Fiber-Bragg-grating based single axial mode Fabry-Perot interferometer and its strain and acceleration sensing applications. J. Lightwave Tech..

[14] Todd M.D., Johnson G.A., Chang C.C. (1999). Passive, light intensity-independent interferometric method for fiber Bragg grating interrogation. Electron. Lett..

[15] Chino S., Ishizawa H., Hosoya S., Koyama S., Fujimoto K. Non-invasive blood pressure measurement—The study of measuring points. Proceedings of the SICE Annual Conference 2016.

[16] Katsuragawa Y., Ishizawa H. Non-invasive blood pressure measurement by pulse wave analysis using FBG sensor. Proceedings of the 2015 IEEE International Instrumentation and Measurement Technology Conference (I2MTC).

[17] Hall J.W., Pollard A. (1993). Near-infrared spectroscopic determination of serum total proteins, albumin, globulins, and urea. Clin. Biochem..

[18] Heise H.M., Bittner A., Marbach R. (1998). Clinical chemistry and near infrared spectroscopy: Technology for non-invasive glucose monitoring. J. Near Infrared Spectrosc..

[19] Malin S.F., Ruchti T.L., Blank T.B., Thennadil S.N., Monfre S.L. (1999). Noninvasive prediction of glucose by near-infrared diffuse reflectance spectroscopy. Clin. Chem..

[20] Kasemsumran S., Du Y., Maruo K., Ozaki Y. (2006). Improvement of partial least squares models for in vitro and in vivo glucose quantifications by using near-infrared spectroscopy and searching combination moving window partial least squares. Chemom. Intel. Lab. Syst..

[21] Koyama S., Morishima M., Miyauchi Y., Ishizawa H. (2013). Non-destructive identification and mixture ratio analysis of cotton-polyester blended textile products by IR spectroscopy. Text. Light Ind. Sci. Technol..

[22] Wold S., Ruhe A., Wold H., Dunn W.J. (1984). The collinearity problem in linear regression, The partial least squares (PLS) approach to generalized inverses. SIAM. J. Sci. Stat. Comput..

[23] Wold S., Sjostrom M., Ericksson L. (2001). PLS-regression, a basic tool of chemometrics. Chemom. Intel. Lab. Syst..

[24] Lorber A., Wangen L.E., Kowalski B.R. (1987). A theoretical foundation for the PLS algorithm. J. Chemom..

[25] Martens H., Neas T. (1989). Multivariate Calibration.

[26] Ahn J.M. (2015). Wave detection in acceleration plethysmogram. Healthc. Inf. Res..

[27] Elgendi M., Norton I., Brearley M., Abbott D., Schuurmans D. (2014). Detection of a and b waves in the acceleration photoplethysmogram. BioMed. Eng. Online.

[28] Sano Y., Kataoka Y., Ikuyama T., Wada M., Imano H., Kawamura K., Watanabe T., Nishida A., Osanai H. (1988). Evaluation of peripheral circulation with accelerated plethysmography and its practical applications report 2. Quantification of inflection points of a waveform. Bull. Phys. Fit. Res. Inst..

